# Tribological Behavior of Carbon-Based Nanomaterial-Reinforced Nickel Metal Matrix Composites

**DOI:** 10.3390/ma14133536

**Published:** 2021-06-24

**Authors:** Amit Patil, Ganesh Walunj, Furkan Ozdemir, Rajeev Kumar Gupta, Tushar Borkar

**Affiliations:** 1 Mechanical Engineering Department, Cleveland State University, Cleveland, OH 44115, USA; a.k.patil@vikes.csuohio.edu (A.P.); g.walunj@vikes.csuohio.edu (G.W.); 2 Department of Materials Science and Engineering, North Carolina State University, Raleigh, NC 27606, USA; fozdemi@ncsu.edu (F.O.); rkgupta2@ncsu.edu (R.K.G.)

**Keywords:** metal matrix composites (MMCs), ball milling, spark plasma sintering, nickel metal matrix nanocomposites, carbon nanotubes, graphene nanoplatelets, tribological behavior

## Abstract

Carbon nanotubes (CNTs) and graphene nanoplatelets (GNPs) with exceptional mechanical, thermal, chemical, and electrical properties are enticing reinforcements for fabricating lightweight, high-strength, and wear-resistant metal matrix composites with superior mechanical and tribological performance. Nickel–carbon nanotube composite (Ni-CNT) and nickel–graphene nanoplatelet composite (Ni-GNP) were fabricated via mechanical milling followed by the spark plasma sintering (SPS) technique. The Ni-CNT/GNP composites with varying reinforcement concentrations (0.5, 2, and 5 wt%) were ball milled for twelve hours to explore the effect of reinforcement concentration and its dispersion in the nickel microstructure. The effect of varying CNT/GNP concentration on the microhardness and the tribological behavior was investigated and compared with SPS processed monolithic nickel. Ball-on-disc tribological tests were performed to determine the effect of different structural morphologies of CNTs and GNPs on the wear performance and coefficient of friction of these composites. Experimental results indicate considerable grain refinement and improvement in the microhardness of these composites after the addition of CNTs/GNPs in the nickel matrix. In addition, the CNTs and GNPs were effective in forming a lubricant layer, enhancing the wear resistance and lowering the coefficient of friction during the sliding wear test, in contrast to the pure nickel counterpart. Pure nickel demonstrated the highest CoF of ~0.9, Ni-0.5CNT and Ni-0.5GNP exhibited a CoF of ~0.8, whereas the lowest CoF of ~0.2 was observed for Ni-2CNT and Ni-5GNP composites. It was also observed that the uncertainty of wear resistance and CoF in both the CNT/GNP-reinforced composites increased when loaded with higher reinforcement concentrations. The wear surface was analyzed using scanning electron microscopy (SEM) and energy dispersive spectroscopy (EDS) analysis to elucidate the wear mechanism in these composites.

## 1. Introduction

Metal matrix composites (MMCs), consisting of a metal matrix reinforced with high-strength ceramic reinforcement, exhibit a very high strength to weight ratio and can be employed at elevated temperatures and severe working conditions due to a synergetic combination of metallic properties, such as high toughness, strength, and ductility, as well as ceramic properties, such as high hardness, high tensile strength, and low coefficient of thermal expansion, with excellent resistance to wear and corrosion. This attractive set of properties with various reinforcing agents and processing routes makes MMCs a favorable material for various applications in the automotive and aerospace industries [1,2,3]. Depending upon the applications and desired properties, the metal matrix composites can be reinforced with several reinforcements, such as metallic carbides, oxides, sulfides, borides, and nitrides [4]. However, the reinforcements should have an excellent arrangement of mechanical, thermal, and chemical properties for structural engineering applications and must possess low density. Furthermore, the increasing demand for improved energy efficiency in almost every engineering application makes it necessary to optimize the material properties with improved tribological performance [5]. Over the past decade, the need for novel material with high-performance surface engineering applications has become the primary enticement for developing carbon-based nanomaterial-reinforced metal matrix composites. Carbonaceous reinforcements, such as carbon nanotubes (CNTs), graphene nanoplatelets (GNPs), and carbon fibers, have attracted substantial attention as a potential solid lubricant reinforcement in the metal matrix owing to their exceptional mechanical, thermal, chemical, and electrical properties [6,7,8]. When reinforced with carbon allotropes, metal matrix composites (MMCs) exhibit higher thermal conductivity, lower coefficient of thermal expansion, and improved wear resistance ability [6,7,8]. These dimensionally and morphologically different reinforcing allotropes of carbon exhibit different strengthening abilities. Carbon fibers exhibit high mechanical and thermal properties, making them suitable for numerous structural applications. However, the anisotropic properties of carbon fibers limit their application as a reinforcing agent in various metal matrices. On the contrary, CNT/GNP-reinforced metal matrix nanocomposites demonstrate isotropic properties, possess a high strength to weight ratio, and exhibit outstanding wear, corrosion, and oxidation resistance. These intrinsic properties make metal matrix nanocomposites reinforced with CNTs/GNPs a latent candidate for numerous high-strength structural and surface engineering applications.

Nickel exhibits numerous attractive properties, such as ductility, toughness, thermal and electrical conductivity, and exceptional resistance to oxidation, wear, and corrosion [4,9]. However, mechanical properties, like strength and tribological properties such as wear resistance, are moderate for a monolithic nickel. Therefore, to improve the properties and compensate for the limitations of monolithic base nickel metal, the nickel metal matrix can be reinforced with various reinforcing agents. Carbonaceous reinforcement, such as CNTs with high aspect ratios and GNPs with a larger surface area, is projected to be an excellent reinforcing material in the ductile nickel metal matrix. Additionally, studies showed enhancement in the mechanical properties of the nickel-based MMCs when reinforced with carbonaceous reinforcement, as pure nickel exhibits a low affinity for carbon and does not form a metastable carbide phase [10,11,12,13]. Additionally, the CNT and GNP reinforcements act as solid lubricants and efficiently reduce the friction coefficient and improve wear resistance [11,14]. Another reason for improved wear resistance of CNT/GNP-reinforced MMCs can be ascribed to the existence of CNT/GNP reinforcement in the metal matrix, which restricts dislocation movement and hinders the plastic deformation of material due to frictional forces [15,16]. Previously, several investigations were carried out to study the effects of solid lubricant reinforcements on the tribological performance and the wear behavior of nickel-based metal matrix composite. Results have demonstrated considerable enhancement in the wear resistance [7,9]. This is most likely due to the frictional reduction of CNTs/GNPs in the metal matrix that resulted in the formation of a lubricious graphitic tribolayer [9,17,18].

Irrespective of the extraordinary strengthening potential and excellent tribological properties of CNTs/GNPS, the utilization of reinforcement to its full capacity is still a major bottleneck due to various difficulties. It is difficult to attain homogeneous dispersion of CNTs/GNPs within the metal matrix due to their agglomeration resulting from strong, cohesive van der Waals forces [19,20,21]. An additional challenge is forming a strong and stable interfacial bonding in conjunction with the retained structure of the carbonaceous reinforcement, which is necessary for effective load transfer from the matrix to the reinforcement. This is necessary for applications where both good tribological properties and high strength are preferred. Furthermore, the selection of reinforcement concentration is a crucial parameter. It can adversely influence the dispersion, affecting the strength enhancement and the tribological behavior of these composites [18,22]. The processing technique is also a considerable factor influencing the porosity levels, dispersion of the reinforcement phase, the interfacial contact between the reinforcement and the metal matrix, and the level of porosity in the microstructure [23,24].

Previously, several attempts were made to efficiently overcome the difficulties involved with the dispersion of CNTs/GNPs in the metal matrix alongside retaining the structural integrity of these carbon-based reinforcements [25,26]. The metal matrix composites reinforced with carbonaceous reinforcement are processed using various processing techniques, such as powder metallurgy, melting and casting, electrochemical deposition, and other novel approaches such as nanoscale dispersion and molecular-level mixing processes. Powder metallurgy is considered a convenient and versatile approach to fabricate near net-shaped bulk composite components with refined microstructure and provide excellent control over the quality and properties of the composites [26,27,28,29,30,31,32]. Previous studies have shown the viability of the ball milling technique to overcome the cohesive van der Waal forces and attain dispersion of carbonaceous reinforcement in the metal matrix. In ball milling, the mixture of metal powder and carbonaceous reinforcement is subjected to repetitive impact forces during milling, which lead to the breakdown of the CNT/GNP agglomeration and increase the interfacial interaction between the reinforcement and metal matrix [33]. Results indicate that materials reinforced with solid lubricants and processed via powder metallurgical technique have extensive surface engineering applications, owing to the improvement in wear resistance and overall mechanical performance [27,28,29]. Alongside the uniform dispersion of reinforcement, strong interfacial interaction between the CNTs/GNPs and the metal matrix, with retained structural integrity, is critical for improving the load-bearing abilities. This improvement is exceptionally crucial for enhancing mechanical properties and the wear resistance of the metal matrix composites reinforced with CNTs/GNPs. Furthermore, the quality of the microstructure also considerably affects the wear resistance of the composites [19,33,34]. Previously, several studies were conducted to utilize ball milling to obtain composite powder and followed by consolidation via the spark plasma sintering (SPS) technique to fabricate metal matrix nanocomposites reinforced with CNTs/GNPs [10,35,36,37]. Spark plasma sintering allows consolidation of the composite powder at a relatively low temperature compared to other sintering techniques. Furthermore, the high heating rate and shorter sintering time in the spark plasma sintering technique inhibit grain growth during the recrystallization process, which improves the wear resistance of the material [38,39,40].

The self-lubricating nature of CNTs/GNPs is often utilized for tribological applications, where high wear resistance and a lower coefficient of friction are desired. These self-lubricating metal matrix composites are typically found in nanocomposite coatings or claddings or bulk metal matrix composite materials. However, these solid lubricant coatings have many limitations, such as poor adhesion, difficulty of replenishment, and relatively shorter life cycle compared to MMCs loaded with carbonaceous reinforcement [14]. Previous research extensively investigated the lubrication effect and the wear resistance of these reinforcements when used as a coating on MMCs [19,41,42,43,44]. However, very limited research has been conducted on utilizing these composites as a reinforcement in a bulk nickel metal matrix to enhance both mechanical properties and the tribological performance of these composites [11,45]. The effects of these reinforcements on the mechanical performance of nickel metal matrix composites are highlighted in our previous research [46,47]. Moreover, the lubricating nature of carbon-based reinforcements in metal matrix composites varies with its morphology. However, very few studies have been conducted to investigate the effect of the geometrically distinct morphology of carbonaceous reinforcements (e.g., carbon nanotubes (CNTs) and graphene nanoplatelets (GNPs)) on the tribological behavior of these composites.

The present research emphasizes the viability of processing carbon nanotube-reinforced nickel metal matrix composites (Ni-CNT) and graphene nanoplatelet-reinforced nickel matrix (Ni-GNP) composites using high-energy ball milling and the SPS technique to obtain a uniform dispersion of carbonaceous reinforcement in the nickel matrix and attain improvement in the tribological behavior. The microstructural characterization and wear testing of the as-processed composites was conducted to investigate the effect of concentration on the dispersion of these carbonaceous nanomaterials in the nickel matrix and the consequent effects on the tribological properties of the composites were investigated. The formation of a wear track due to friction and the wear mechanisms involved are also discussed.

## 2. Materials and Methods

### 2.1. Experiment Methods

**Figure 1:**
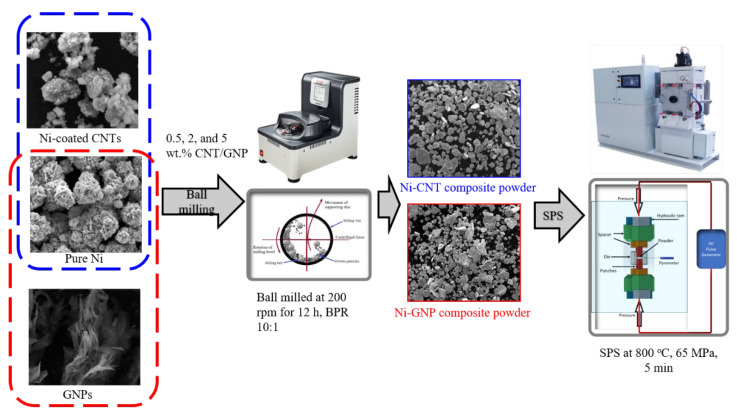
Schematics for processing of Nickel-Carbon nanotubes (Ni-CNT)/ Nickel-Graphene nanoplatelets (Ni-GNP) composites.

The elemental spherical nickel powder (5–7 μm, 99.9% purity) was procured from Alfa Aesar (Tewksbury, MA, USA), whereas the Ni-coated CNTs and raw GNPs were procured from Nanostructured & Amorphous Materials, Inc. (Katy, TX, USA). The schematics for the processing of Ni-CNT/GNP composites is shown in Figure 1. The powder mixture of pure nickel and 0.5, 2, and 5 wt% of multi-walled carbon nanotubes (referred to as Ni-0.5CNT, Ni-2CNT, and Ni-5CNT) was used to obtain the Ni-CNT nanocomposite powder. Nickel-coated multi-walled carbon nanotubes (Ni: ~60 wt%, CNTs: ~38 wt%) instead of raw CNTs were used to obtain a uniform dispersion of CNTs in the nickel matrix. The Ni-CNT nanocomposite powder was obtained via the ball milling technique employed in a Planetary micro mill P7 premium line (FRITSCH, Idar-Oberstein, Germany). The dry ball milling was carried out in an argon atmosphere with a ball to powder weight ratio (BPR) of 10:1 at a milling speed of 200 rpm for 12 h. The as-processed Ni-CNT composite powder was sintered via the spark plasma sintering technique (SPS 10-3, Thermal Technology LLC, Santa Rosa, CA, USA) at a temperature of 800 °C under 65 MPa pressure and a sintering duration of 5 min.

A similar processing technique was utilized for the fabrication of graphene nanoplatelet-reinforced nickel metal matrix composites with varying weight fractions (0.5, 2, and 5 wt%) of the GNP reinforcement (referred to as Ni-0.5GNP, Ni-2GNP, and Ni-5GNP). In Ni-GNP composites, raw GNPs, unlike in Ni-CNT composites, where nickel-coated CNT reinforcement was utilized. Table 1 summarizes the properties of multi-walled carbon nanotubes and graphene nanoplatelets [7,33,48,49,50,51,52,53,54,55,56].

### 2.2. Characterization Techniques

The SPS-processed pure nickel and Ni-CNT and Ni-GNP composite samples were mounted using graphite-based conductive mounting powder (Allied High Tech Products, Inc, Los Angeles, CA, USA) and polished using silicon carbide papers (BUEHLER, Lake Bluff, IL, USA) with increasing grit sizes. The final metallographic finish was obtained via polishing with a 0.04 µm colloidal silica suspension on a microcloth for characterization. A scanning electron microscope (SEM) (Inspect F50, FEI Corporation, Hillsboro, OR, USA) was utilized to obtain micrographs and study the effects of CNT/GNP concentration on dispersion and the microstructure of the composites. The Horiba XploRA PLUS confocal Raman microscope (HORIBA, Kyoto, Japan) in combination with LabSpec 6 software (2019, Hillsboro, OR, USA) was employed to perform Raman spectroscopy characterization. The spectra were acquired with a laser excitation wavelength of 532 nm and a spatial resolution of 0.5 μm. The instrument was calibrated using a crystalline Si wafer (520.6 cm^−1^ Raman active peak) before testing. Vickers microhardness was obtained using a Wilson VH1202 hardness tester by BUEHLER (Lake Bluff, IL, USA), with an accuracy of ±1.5%. The Vickers microhardness value of Ni-CNT and Ni-GNP composites was evaluated under a load of 0.5 N with a dwell of 10 s, as recommended by ASTM E384 standards [57]. A pyramid-shaped diamond indenter was utilized for the indentation during measurements. An average value of at least ten microhardness was taken into consideration. The wear test was performed with a ball-on-disc tribometer. The test was conducted at room temperature, and the samples were exposed openly in lab air (~40% Relative humidity) during the tests. The Nanovea T50 tribometer (Irvine, CA, USA) was employed, and the tests were performed under a normal force of 1 N with a 3 mm diameter Si_3_N_4_ ball with a microhardness value of ~1620 HV. The test was carried at a constant sliding velocity of 50 mm/s with a track radius of 2 mm for 10,000 unidirectional revolutions. At least three wear tests were carried out to determine the coefficient of friction. The wear tracks were examined using the Energy dispersive spectroscopy (EDS) technique to observe the features of the wear tracks and elucidate the wear phenomena.

## 3. Results and Discussion

### 3.1. X-ray Diffraction (XRD) Analysis

**Figure 2:**
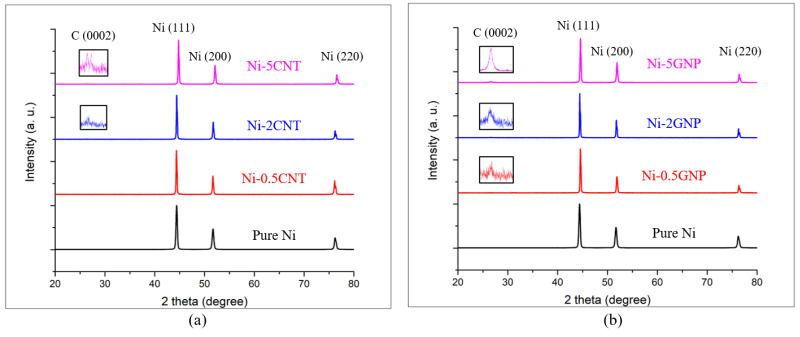
X-ray diffraction (XRD) pattern for (**a**) pure Ni and Ni-CNT composites; (**b**) pure Ni and Ni-GNP composites.

X-ray diffraction patterns of pure Ni, Ni-CNT and Ni-GNP composites are shown in Figure 2a,b. The XRD pattern exhibits the peaks associated with (111), (200), and (220) crystallographic planes of face centered cubic (FCC) nickel, and the corresponding two-theta degree positions of the peaks are in accordance with the XRD data files of pure nickel [58]. The peak related to hexagonal closest packed (HCP) carbon (0002) is observed in Ni-2CNT, and Ni-5CNT composites (shown in magnified view as an inset in Figure 2), exhibit increasing intensity with increasing CNT concentration. However, peaks related to carbon are not visible in the Ni-0.5CNT composite sample, mainly owing to the relatively lower content of CNTs in these composites, which is beyond the detectable resolution of XRD. A similar pattern was visible in Ni-GNP composites, and peaks corresponding to crystallographic planes of FCC nickel and HCP carbon are the only peaks visible in the XRD pattern in SPS-processed pure Ni and Ni-GNP composites. In both Ni-CNT and Ni-GNP composites, the absence of any other peak, primarily Ni_3_C, in these XRD patterns qualitatively implies that no reaction between the nickel and CNTs occurred to form the usually detrimental carbide phase during the ball milling and spark plasma sintering process.

### 3.2. Microstructural Analysis

**Figure 3:**
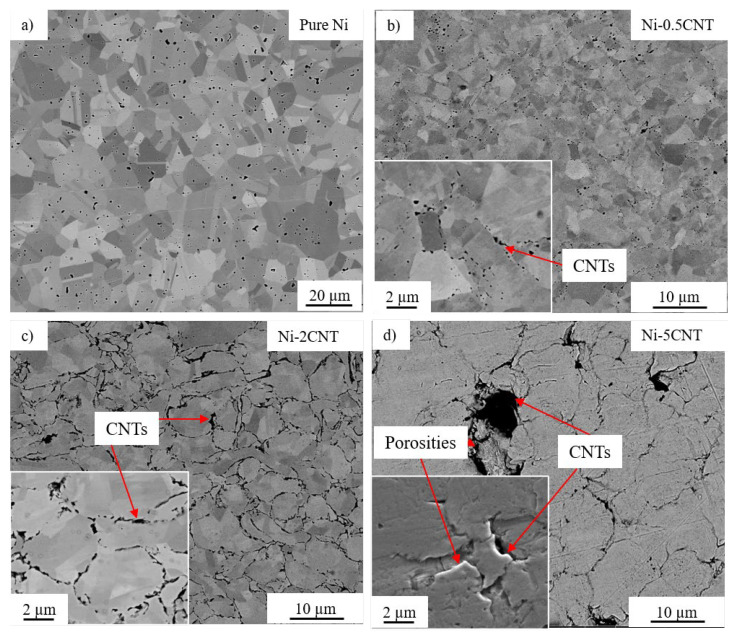
SEM micrographs of (**a**) pure Ni, (**b**) Ni-0.5CNT, (**c**) Ni-2CNT and (**d**) Ni-5CNT composite.

**Figure 4:**
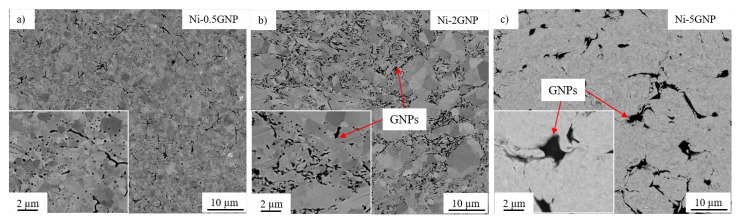
SEM micrographs of (**a**) Ni-0.5GNP, (**b**) Ni-2GNP, (**c**) Ni-5GNP composite.

SEM micrographs of the SPS-processed pure Ni and Ni-CNT composites are shown in Figure 3. Pure nickel exhibited a uniform microstructure with randomly oriented grains. All the Ni-CNT composites exhibited significant grain refinement as compared to the pure nickel sample. This can be attributed to the mechanical milling and the existence of CNTs in the nickel metal matrix, which act as a pinning point and inhibit the grain growth during recrystallization in SPS processing. Figure 3 reveals the dispersion of CNTs throughout the nickel matrix, and the CNTs are predominantly situated along the nickel grain boundaries. The SEM micrographs for Ni-GNP composites shown in Figure 4 reveal a similar pattern. In both Ni-CNT and Ni-GNP composites, it is noted that the dispersion of CNTs/GNPs and the size of clusters vary with the reinforcement concentration in these composites. The size and number of CNT clusters increase with an increasing weight fraction of CNTs. The Ni-5CNT and Ni-5GNP composites exhibited the most prominent clusters and inferior microstructure with porosity surrounding the CNT/GNP clusters. This is mainly due to the existence of a relatively higher content of CNTs in these composites, which leads to increased agglomeration. It is apparent that the nickel grain size decreases, and the size of the CNT clusters increases as the CNT concentration increases from 0.5 to 2 wt%. However, as the CNT concentration increases further, up to 5 wt%, the CNT clusters increase in size with relatively larger grain size and increased porosity. This is primarily due to the inhomogeneous dispersion of CNTs in the nickel matrix. Similarly, in Ni-GNP composites, as the GNP content in these composites increases further, beyond 2 wt%, porosities are observed in the composite. The Ni-5GNP composite exhibited large GNP clusters with the presence of porosity around the inhomogeneously dispersed large GNP clusters.

### 3.3. Raman Spectroscopy Analysis

**Figure 5:**
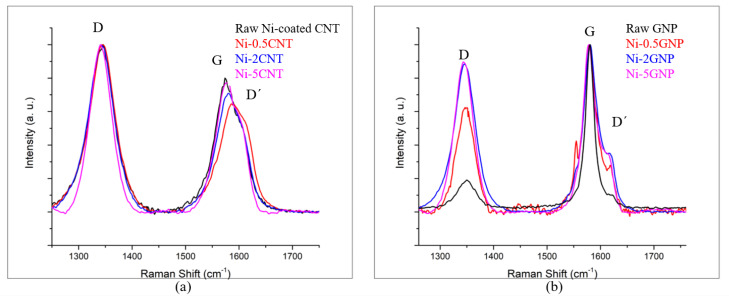
Raman spectra for (**a**) raw Ni-coated CNTs and Ni-CNT composites; (**b**) raw GNPs and Ni-GNP composites.

Raman spectroscopy analysis was performed to determine the structural changes/defects in the CNTs/GNPs that transpired during ball milling as well as those due to heat and pressure during SPS processing. The Raman spectra obtained for raw Ni-coated CNTs and raw GNPs, as well as the SPS-processed Ni-CNT and Ni-GNP composites with varying reinforcement content (0.5, 2, 5 wt%), are shown in Figure 5. The Raman spectra for raw Ni-coated CNTs and raw GNPs with primary peaks at ~1340, ~1580, and ~1620 cm^−1^ Raman shift wavenumbers, corresponding to the D, G, and D′ peaks, respectively, serve as a reference for the comparison [11,17,59,60]. The structural disorder induced in the carbon allotropes can be assessed in a qualitative sense via the peak position, their intensities, and the intensity ratio of the D to G peak (I_D_/I_G_). The D peak, which occurred due to the breathing modes of *sp^2^* bonded carbon atoms, is associated with the defects induced in the structure, while the G peak corresponds to the C–C in-plane bond stretching of all the pairs of *sp^2^* bonded carbon atoms in the rings and chains. The D′ peak is associated with the double resonance band induced due to disorders. These characteristic peaks are deemed as defect-dependent structural indicators and often depict changes in peak position and the intensity ratio depending upon the structural changes induced during processing [59,60,61,62]. Compared with the raw nickel-coated CNTs, the Ni-CNT composites also exhibit characteristic D, G, and D′ peaks corresponding to CNTs, validating the presence of CNTs in these composites. The Raman spectra for Ni-CNT composites shown in Figure 5a reveal a few trivial peak changes in these composites after processing, which qualitatively indicate that no severe structural disorders were induced during processing. The G peak position is shifted in all Ni-CNT composites compared to raw Ni-coated CNTs. However, the peak shift is decreased as the CNT concentration increases from 0.5 to 5 wt%. The defect index, indicated by the intensity ratio I_D_/I_G_, slightly increases in all Ni-CNT composite samples, implying a slightly disordered form of carbon. However, the difference is very minimal in the Ni-5CNT composites, which is attributed to the higher concentration of carbonaceous reinforcement. These trends in the Raman spectra may indicate a minor structural disorder induced while processing these composites. They also reveal that as the CNT concentration in these composites is increased from 0.5 to 5 wt%, the structural disorder induced in the CNTs is reduced. This indicates that at higher CNT concentrations, the structural integrity of CNTs is relatively well preserved during ball milling followed by SPS processing.

Figure 5b shows the Raman spectra for raw GNPs and SPS-processed Ni-GNP composite samples. The Raman spectra exhibit D, G, and D′ peaks associated with carbon with no other peak corresponding to any interfacial metal carbide, implying that no metastable carbide phase formed during processing [63,64]. The Raman spectra show the relatively higher peak shift of the D peak, a disordered GNP vibrational peak, for Ni-0.5GNP composites compared with Ni-2GNP and Ni-5GNP composites, with slight structural defects induced in the GNPs in Ni-0.5GNP. It is likely that when a lower concentration of reinforcement is present in the composites, the possibility of damaging the structural integrity of reinforcement during processing increases [65,66]. Another pattern observed in the intensity ratio (I_D_/I_G_) for these composites is that the intensity ratio is lower for Ni-0.5GNP composite, whereas the Ni-2GNP and Ni-5GNP composites exhibited relatively higher intensity ratios. This contradicts the observation made for CNT-reinforced composites, where a higher intensity ratio is observed for Ni-0.5CNT composite, implying a higher order of disorder in the CNT structure. In Ni-0.5GNP composite, the relatively lower intensity ratio could be associated with the phenomena linked to I_D_/I_G_ exhibiting two distinct defect behaviors in graphene. In the low defect density regime, the I_D_/I_G_ increases with increasing disorders up to the high defect density regime. After this stage, a transition in the pattern occurs, and the intensity ratio decreases due to the higher defect density, which essentially engenders a more elastic scattering of the spectra [67]. It can be said that this increasing defect density is possibly due to an amorphous carbon structure, whereas the low defect density regime occurs due to a nanocrystalline graphitic structure [68,69].

Furthermore, the intensity of the D peak is directly proportionate to the defect level in the composite samples. The D band is usually weak in GNPs compared to carbon nanotubes [70,71]. As the CNTs comprise graphene sheets rolled into a concentric cylindrical form, their Raman spectra depict disorders similar to defective pyrocarbons, and the peak associated with edge-induced structural defects (i.e., D band) exhibited the highest intensity [72,73], thus possibly indicating that more defects are present in the CNT-reinforced metal matrix composite systems. Additionally, the intensity ratio (I_D_/I_G_) is slightly increased compared with raw, pristine GNPs. This is mainly a disordered form of carbon on the surface of the GNPs due to impact forces during high-energy ball milling. However, no peak shift is observed in the spectra, which indicates that the structural integrity of GNPs in these composites is relatively well preserved after processing.

### 3.4. Microhardness

Vickers microhardness of SPS-processed pure nickel and Ni-CNT and Ni-GNP composites is listed in Table 2. Pure nickel exhibited a microhardness value of around ~112 HV. It is observed that the microhardness of all Ni-CNT and Ni-GNP composites increased substantially as compared to the SPS-processed pure nickel, mainly due to the incorporation of carbonaceous reinforcement in the nickel matrix adjunct with significant grain refinement of the nickel matrix. This is mainly due to the existence of carbonaceous nanomaterials, inhibiting the grain growth of the nickel matrix in adjunct with high-energy impact forces during ball milling. The refined microstructure of Ni-CNT and Ni-GNP composites is also visible from the SEM micrographs shown in Figure 3 and Figure 4. Ni-0.5CNT composites exhibited the highest microhardness value of ~187 HV compared with other Ni-CNT composites. It is ascribed to the uniform dispersion of CNT reinforcement within the refined nickel metal matrix. It is noticed that as the concentration of the reinforcement in these composites increases the microhardness value decreases. The Ni-5CNT composites exhibited the lowest microhardness as compared to other Ni-CNT composites, mainly due to the existence of large CNT clusters within the nickel matrix. Furthermore, a significant deviation in the microhardness value has been observed in Ni-5CNT composites. This is mainly due to inhomogeneous CNT dispersion, leading to large CNT clusters and grain size distribution and inferior nickel matrix microstructure with the presence of porosity around the CNT clusters. In Ni-GNP composites, the Ni-0.5GNP composites exhibited the highest microhardness value of ~215 HV, primarily due to homogeneously dispersed GNPs in the nickel matrix and substantial grain refinement of the nickel metal matrix. The relatively smaller grain size of the Ni-0.5GNP composite is also evident from the SEM micrograph shown in Figure 4. As the concentration of GNP reinforcement in these composites increases, in the case of Ni-2GNP and Ni-5GNP composites, a decrease in microhardness was observed, which can be ascribed to large GNP clusters distributed across the nickel matrix. Out of all these composites, GNP-reinforced nickel matrix composites exhibited relatively higher microhardness than that of their Ni-CNT counterparts. This discrepancy, irrespective of the composites reinforced with an equivalent quantity of reinforcement in the nickel matrix, is possibly due to the 2D structure of GNPs exhibiting more interaction with the matrix grains and making it more effective in hindering plastic dislocation during deformation. Furthermore, the microhardness values obtained for other nickel MMCs reinforced with different carbonaceous reinforcements with varying concentrations and processed via different techniques are also listed in Table 2. They serve as a benchmark to comprehend the overall improvement in the microhardness attained for Ni-CNT/GNP composites processed in this research work. The results are in good agreement with other nickel MMCs reinforced with different carbonaceous reinforcements and varying concentrations, implying potential applications for them as processed composites.

### 3.5. Tribological Performance

**Figure 6:**
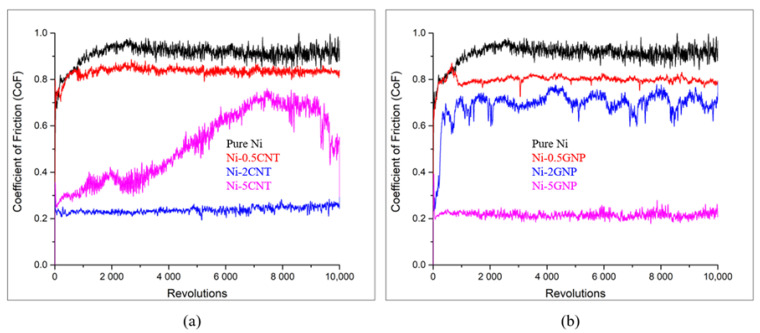
Coefficient of friction plots for (**a**) pure Ni and Ni-CNT composites; (**b**) pure Ni and Ni-GNP composites.

The plots representing the coefficient of friction (CoF) as a function of revolution for the SPS-processed pure nickel in contrast with Ni-CNT and Ni-GNP composites are shown in Figure 6. The ball-on-disc technique, which is a sphere-on-disc type of wear test exhibiting a point contact at which shear stress is applied parallel to the mating surface, was utilized to investigate the wear under sliding friction. Results indicate that the presence of CNT/GNP reinforcement in these composites was advantageous in lowering the CoF in contrast with the SPS-processed pure nickel. Pure nickel demonstrated the highest CoF value of ~0.9. In contrast, the relatively lower CoF values were obtained for Ni-CNT composites, primarily due to the presence of carbonaceous reinforcement essentially acting as a solid lubricant during friction. The improvement in the wear behavior of these composites can also be ascribed to the grain refinement of the nickel matrix. It is often observed that the wear resistance of the material improves with increasing microhardness [78]. From Figure 6a, it is observed that Ni-0.5CNT composites exhibited the highest CoF value of ~0.8 in comparison with other Ni-CNT composites. This is primarily due to the relatively lower weight fraction of CNTs. The lowest CoF of ~0.22 was observed for Ni-2CNT composites, which can be ascribed to the high CNT concentration coupled with considerably better dispersion, which is also visible from the SEM micrographs (Figure 3). Irrespective of the higher concentration of CNTs in Ni-5CNT composites, they exhibited a higher CoF compared to the Ni-2CNT composite. This essentially indicates that, along with the concentration of CNT reinforcement, other factors such as deagglomeration and dispersion in the metal matrix, as well as the grain size of the metal matrix, also play a critical role in enhancing the overall tribological behavior of these composites. The Ni-5CNT composites exhibited a lower CoF of ~0.35 during the initial cycles. However, with the increasing sliding distance, the CoF value increased. In contrast, for other Ni-CNT composites, a steady CoF was observed throughout the test run. The possible reasons for this unusual frictional behavior in Ni-5CNT can be attributed to the inhomogeneous dispersion of CNT clusters and the nickel grain size distribution. Another possible reason for this wear trend could be associated with the inferior quality of the nickel metal matrix, with porosities around agglomerated CNTs and microstructural defects, which are also evident from the SEM micrographs obtained via a secondary electron detector (shown as an inset in Figure 3). This may be due to the agglomeration of CNTs in the matrix, interrupting the diffusion bonding among the composite powder during sintering via the SPS process. This resulted in inhomogeneous dispersion with inferior microstructure and increased porosities. This is also supported by the higher variation observed in the microhardness of Ni-5GNP composites, as shown in Table 2. However, a detailed investigation is required to understand tribological behavior and potential wear mechanisms in these high-concentration CNT-reinforced composites. A similar trend in the CoF variation was reported in bulk CNT–nickel composite fabricated via a different technique [15]. Beyond a certain critical concentration of CNT reinforcement in these composites, the CoF and wear loss increase. Similar observations were made in CNT-reinforced aluminum metal matrix composites. These composites exhibited relatively lower CoF up to a ~5 vol% concentration, and as the CNT concentration further increased beyond 5 vol%, the CoF and the wear loss increased [79].

In Figure 6b, a small bump is observed during the initial test revolutions for the Ni-0.5GNP composite. During the initial 1000 revolutions, the CoF increased up to ~0.85 and later exhibited a steady CoF of ~0.78 through the entire test run. This could be associated with the adhesive wear between the nickel metal matrix and the Si_3_N_4_ counterface during sliding before interacting with lubricious GNPs. This resulted in significant plastic deformation during the initial cycles until a lubricant layer was formed by sheared GNPs, which reduced the plastic deformation and further stabilized the CoF during the rest of the test. Another possible reason could be surface irregularities, which increased the CoF until a lubricious layer was formed due to the presence of GNPs on the wear track. This phenomenon is usually witnessed in the case of monolithic metals or composites reinforced with a very minimal concentration of reinforcements [78,80,81]. In Ni-GNP composites, the wear trend indicates that as GNP content increases in these composites, the CoF decreases. Unlike in Ni-CNT composites, the Ni-5GNP composite exhibited the lowest CoF of ~0.2. However, a fluctuation in CoF of ~0.6 to ~0.75 over the test run was observed in Ni-2GNP composites, whereas, irrespective of the higher concentration of GNPs and evident porosities in the Ni-5GNP composite, it exhibited a steady CoF of ~0.2 throughout the test cycles. The fluctuation in CoF could possibly be due to surface irregularities on the specimen and inhomogeneous GNP dispersion near the test vicinity. It may be associated with the relatively larger wear track width on the Ni-2GNP composite, which is indicative of extensive abrasive and adhesive wear. Similar observations were made for CNT- and GNP-reinforced Al_2_O_3_ composites [82]. It was observed that the CNTs/GNPs are prone to agglomeration due to their high aspect ratio/surface area, and thus deteriorate the lubricating effect and uniformity of the wear resistance in the nanocomposites [83,84]. Another reason could be the accumulation of wear debris from worn-off grains on the wear track, which increases local adhesion between sliding surfaces and leads to fluctuations in the CoF [85,86,87]. However, this requires further investigation.

**Figure 7:**
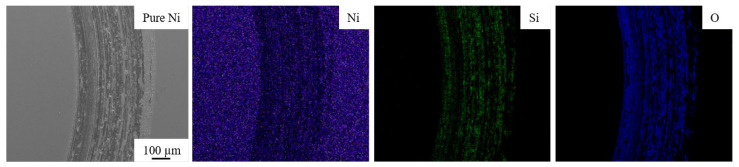
Energy dispersive spectroscopy (EDS) maps of wear track on pure Ni.

**Figure 8:**
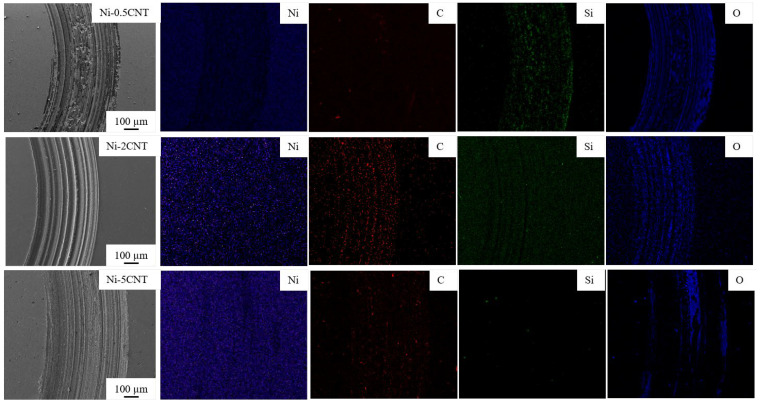
EDS maps of wear track on Ni-CNT composites.

**Figure 9:**
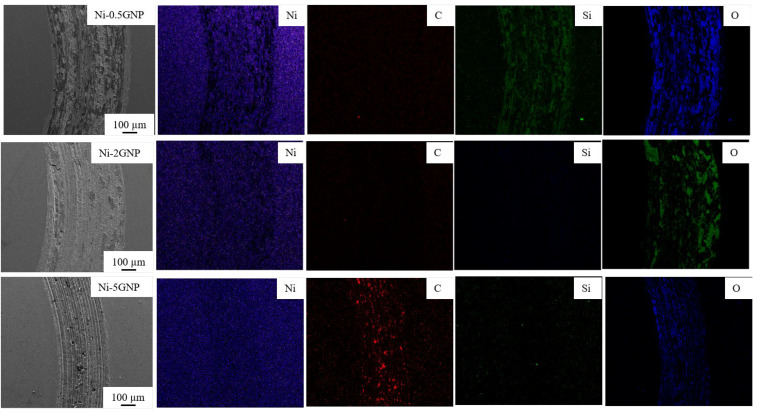
EDS maps of wear track on Ni-GNP composites.

To further elucidate the wear mechanism and understand the tribological behavior involved, the SEM micrographs and corresponding elemental maps were obtained using the EDS technique. SEM micrographs, and elemental EDS map of the wear track, for pure nickel and Ni-CNT and Ni-GNP composites, are shown in Figure 7, Figure 8 and Figure 9. The SEM micrographs of the wear track of pure nickel (Figure 7) reveal a grinding structure along the direction of frictional sliding. The existence of fine grooves in the wear track implies that an abrasive wear mechanism occurs in both SPS-processed pure nickel as well as in Ni-CNT/GNP composites. The EDS map of the wear track on pure nickel evidently reveals the presence of the highest content of oxygen and silicon on the wear track. The formation of oxide wear debris indicates that oxidation wear is involved. The EDS maps of pure Ni are shown in Figure 7 and those of Ni-CNT/GNP composites are shown in Figure 8 and Figure 9 and reveal the existence of a transfer layer of an oxide material transferred from the Si_3_N_4_ counterface. This is possibly due to the heat generated under the frictional force during sliding, which causes the thermal oxidation of the worn-out debris to form a transfer layer of SiO_2_ and possibly NiO_2_. However, the formation of NiO_2_ is considered beneficial in some cases, as it acts as a protective layer and reduces the wear rate [43,88]. Furthermore, in comparison to pure nickel, relatively smaller traces of silicon are apparent from the EDS maps of wear tracks of CNT/GNP-reinforced composite samples shown in Figure 8 and Figure 9. This is primarily ascribed to the presence of carbonaceous reinforcement, forming a protective lubricant layer on the wear track and reducing the CoF and oxidation wear in the composites. It also diminishes the relative wear of the Si_3_N_4_ counterface, which reduces the oxidation and formation of SiO_2_ during interfacial sliding. From Figure 8 and Figure 9, it is observed that the nickel matrix reinforced with 0.5 wt% of CNT/GNP reinforcement displayed wear track similar to pure nickel. However, the traces of silicon and oxygen on the wear track are significantly reduced as the content of reinforcement in these composites is increased. The traces of carbon are distinctly visible on the interior of the wear tracks on composites reinforced with a higher concentration of CNTs/GNPs (2 and 5 wt%). This may be ascribed to the interfacial sliding between the composite and the counterface, resulting in the plowing and delamination of CNTs/GNPs located in the contact zone. These pulled-out reinforcements are sheared and result in degradation and graphitization of CNTs/GNPs in the contact zone. Under sliding forces, the CNTs and GNPs located around the grain boundaries in the contact zone are squeezed out from the nickel matrix and are subjected to graphitization to form a lubricious tribolayer on the wear track.

## 4. Conclusions

In this research, nickel metal matrix composites reinforced with carbon nanotubes (CNTs) and graphene nanoplatelets (GNPs) were successfully fabricated via ball milling followed by the spark plasma sintering (SPS) technique. The variation in the reinforcement concentrations in these composites demonstrated disparity in the dispersion, microhardness, and tribological behavior of these composites. The results revealed that the increasing concentration of carbonaceous reinforcement in these composites resulted in a lowered coefficient of friction due to the formation of a lubricious carbon-rich tribolayer on the sliding interface. Ni-2CNT and Ni-5GNP composites exhibited the lowest CoF as compared with other CNT/GNP-reinforced counterparts with relatively lower reinforcement concentrations. The overall trend indicates that with an increasing concentration of reinforcement in these composites, the dispersion and grain refinement of the metal matrix are affected significantly, which resultantly affects the microhardness, wear resistance, and coefficient of friction of these composites. The present study emphasizes the potential of CNT/GNP reinforcement as a solid lubricant in nickel metal matrix composites for high-temperature surface engineering applications.

## Figures and Tables

**Table 1:** Properties of Carbon nanotubes (CNT) and Graphene nanoplatelets (GNPs) [7,33,48,49,50,51,52,53,54,55,56].

Properties	CNT	GNP
Dimensions	Inner Diameter: 3–5 nm Outer Diameter: 8–15 nm Length: ~50 μm	Lateral: 5–10 μm Thickness: 4–20 nm No. of layers: <20
Elastic moduli	~1 TPa	
Density	~1.8–2.2 g/cm^3^	
Tensile strength	~150 GPa	~130 GPa
Thermal conductivity	3500–6000 Wm^−1^K^−1^	4800–5300 Wm^−1^K^−1^

**Table 2:** Vickers microhardness value for pure Ni and Ni-CNT and Ni-GNP composites and other nickel MMCs with carbonaceous reinforcement from the literature.

Sr. No.	Composites	CNT/GNP (wt%)					References
		0	0.5	1	2	5	
1.	Ni-CNT	112 ± 2	186 ± 2	-	178 ± 2	132 ± 7	Present research
2.	Ni-GNP	112 ± 2	215 ± 2	-	185 ± 3	124 ± 4	Present research
3.	Ni-CNT	140	219	203.5	-	-	[74]
4.		115	145	-	-	-	[75]
5.		107.1	-	121.3	123.9	135.1	[76]
6.		77.52	-	94.5 (5 vol%)	-	-	[17]
7.		77.52	-	98.6 (5 vol%)	-	-	
8.	Ni-GNP	77.52	170 (2.5 vol%)	150 (5 vol%)	-	-	[10]
9.	Ni-Gr	164	-	79	-	-	[77]

## Data Availability

The data presented in this study are available on request from the corresponding author.
